# Electrochemical Potential Gradient as a Quantitative in Vitro Test Platform for Cellular Oxidative Stress

**DOI:** 10.3390/antiox5030023

**Published:** 2016-07-11

**Authors:** Carson Bryant, Donald Atha, Vytas Reipa

**Affiliations:** Biosystems and Biomaterials Division, National Institute of Standards and Technology, Gaithersburg, MD 20899, USA; carson.j.bryant@vanderbilt.edu (C.B.); Donald.atha@nist.gov (D.A.)

**Keywords:** cellular oxidative stress, electrochemical measurements, cultured Chinese Hamster Ovary cells, redox potential gradient

## Abstract

Oxidative stress in a biological system is often defined as a redox imbalance within cells or groups of cells within an organism. Reductive-oxidative (redox) imbalances in cellular systems have been implicated in several diseases, such as cancer. To better understand the redox environment within cellular systems, it is important to be able to characterize the relationship between the intensity of the oxidative environment, characterized by redox potential, and the biomolecular consequences of oxidative damage. In this study, we show that an in situ electrochemical potential gradient can serve as a tool to simulate exogenous oxidative stress in surface-attached mammalian cells. A culture plate design, which permits direct imaging and analysis of the cell viability, following exposure to a range of solution redox potentials, was developed. The in vitro oxidative stress test vessel consists of a cell growth flask fitted with two platinum electrodes that support a direct current along the flask bottom. The applied potential span and gradient slope can be controlled by adjusting the constant current magnitude across the vessel with spatially localized media potentials measured with a sliding reference electrode. For example, the viability of Chinese Hamster Ovary cells under a gradient of redox potentials indicated that cell death was initiated at approximately 0.4 V vs. standard hydrogen electrode (SHE) media potential and this potential could be modified with antioxidants. This experimental platform may facilitate studies of oxidative stress characteristics on different types of cells by enabling imaging live cell cultures that have been exposed to a gradient of exogenous redox potentials.

## 1. Introduction

Oxidative stress (OS) in biological systems is defined as an imbalance between reactive oxygen species (ROS) and the organism’s antioxidant defenses. ROS plays a critical role in cellular processes, such as signaling, gene activation and defense against foreign organisms [1]. An imbalance in ROS regulation leads to the disruption of the cellular redox homeostasis, oxidative damage to DNA, proteins and lipids [2] and potential disease such as cancer. The ubiquitous nature of ROS effects in biological systems has suggested the need for studies into their measurements and their mode of action [3,4]. One metric of the relative strengths of oxidizers is the effective redox potential experienced by the cells. Establishing the relationship between the intensity of the exogenous oxidative action, characterized by the external redox potential, and the biomolecular consequences that can occur inside of cells is critical to elucidating how oxidative effects influence biological systems [5].

Oxidative stress experiments in the lab environment require subjecting biological systems to a given level of oxidative stress that is biased relative to the system’s natural equilibrium. Popular in vitro OS inducers include exogenous chemical agents, such as hydrogen peroxide, paraquat, menadione, *t*-butyl hydroperoxide, ionizing radiation or heat shock [6,7]. However, the oxidizing strength of these methods and reagents can be difficult to quantify due to variations in expressing oxidant concentration and to subsequently describe in thermodynamic terms, such as formal potential values. The effect of OS can be defined by quantifying internally produced ROS, oxidative damage markers or by evaluating the mobilization of the cell’s antioxidant activity [8]. For example, by measuring the cytosolic concentrations of reduced and oxidized glutathione [9,10] or other electrochemical couples [9] the intracellular redox potential can be calculated. Such data then allows the establishment of the relationship between the biochemical effects and the thermodynamic and kinetic models, even if, strictly speaking, the biological systems do not operate in equilibrium. In addition to disrupting redox homeostasis, each of the redox agents may also have a specific chemical interaction with cell components, which have to be accounted for during interpretation of the OS experiments [10]. Spontaneous decomposition of ROS initiators such as hydrogen peroxide or *t*-butyl hydroperoxide is strongly affected by culture media composition, in particular, the presence of transition metal ions [11], storage history and exposure to air oxygen [4,12]. Moreover, the relationship between the agent activity and equivalent redox potential, as experienced by the cell in vitro, is complex and may not be easily calculated [13]. Similar issues are encountered when cells are subjected to an ionizing radiation exposure. Here, ROS can be generated due to water or oxygen radiolysis or direct photonic attack on DNA, proteins and lipids, and both processes contribute to the cellular redox state [14,15]. Equivalent radiation doses can result in substantially different oxidative stress levels and cytotoxicity, depending on the experimental setup, thus obstructing data comparison across laboratories and hampering interpretation.

Oxidative stress has been shown to be the main mechanistic paradigm of nanoparticle induced toxicity [16]. Exposure to oxidizing substrates, such as some oxide nanoparticles, induces an imbalance of the intracellular redox state, leading to inflammation and cytotoxic effects. A theoretical framework, linking nanoparticle electronic structure with the standard redox potentials of the biological systems (estimated to cover the potential range from −0.35 V to 0.37 V vs. standard hydrogen electrode (SHE) [17]) was offered [16] and later validated experimentally [18]. Electrochemical methods in biological systems are often used for the detection of various ROS species during the cellular redox processes [19], yet are rarely used for inducing redox potentials on cells [20]. Electrochemical setups have been extensively applied to impose an electric field in galvanotaxis experiments [21,22] in cell culture systems. However, imposing the desired electrochemical potential value at the particular media location is more challenging, as it depends on the distance from electrodes, electrode geometry and the media conductivity [23].

**Figure 1:**
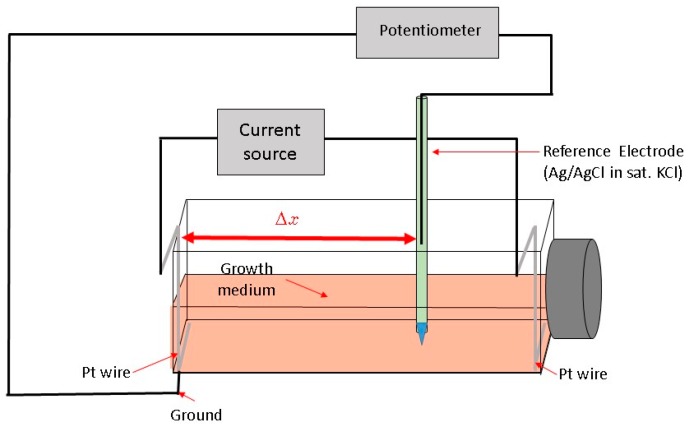
Schematic view of the cell growth flask during potential calibration (see text).

Here, we describe a novel way to create a redox environment for surface-attached mammalian cells by exposure to an electrochemical potential gradient. With our experimental approach we can impose a stable, well-controlled linear potential profile over a cell monolayer and as a result observe a cellular response to a gradient-generated exogenous redox environment. The bipolar electrode arrangement setup [23,24,25,26] was adapted for the rectangular cell growth flask (Figure 1).

A constant current between two Pt wire electrodes in cell medium generates an electric field and a linear potential gradient over the surface attached cell monolayer. The potential span and gradient slope in the medium along the flask bottom can be controlled by adjusting the current magnitude and the spatial location-potential relation is calibrated with a reference electrode, mounted on a translator stage. Surface attached mammalian cells are exposed to a linearly varying electrochemical potential that depends on their location along the flask bottom and the distance from the grounded electrode (Δx). Subsequently, we measure cell viability gradients within an established range of electrochemical treatment levels and relate the cell viability to the specific media electrochemical potential. Cells subjected to increasingly higher oxidative potentials undergo cell death, as confirmed by live/dead fluorescent probe emission profiles.

## 2. Experimental Section

### 2.1. Materials

Hydrogen peroxide (30%, American Chemical Society grade) was purchased from J.T. Baker (Center Valley, PA, USA) and stored at 4 °C and the l-Glutathione, reduced (GSH) was from Sigma-Aldrich (St. Lois, MO, USA). Stock cultures of Chinese Hamster Ovary cells, CHO K1 (ATCC, Manassas, VA, USA) were grown at 37, 5% CO_2_ and 90% relative humidity in Dulbecco’s Modified Eagle’s Medium (Gibco, Carlsbad, CA, USA), 10% (vol. frac.) fetal bovine serum, FBS (Gibco), 1% (*v*/*v*), penicillin-streptomycin (100 units/mL, and 100 mg/mL). Rectangular cell growth flasks (SlideFlask, Nunc A/S, Roskilde, Denmark, 18 mm by 46 mm) were fitted out with 0.5 mm Pt wire electrodes, placed along both lower edges of the bottom wall. Initially 3.8 × 10^5^ cells per flask were seeded in 5 mL of media.

### 2.2. Electrochemical Treatment

**Figure 2:**
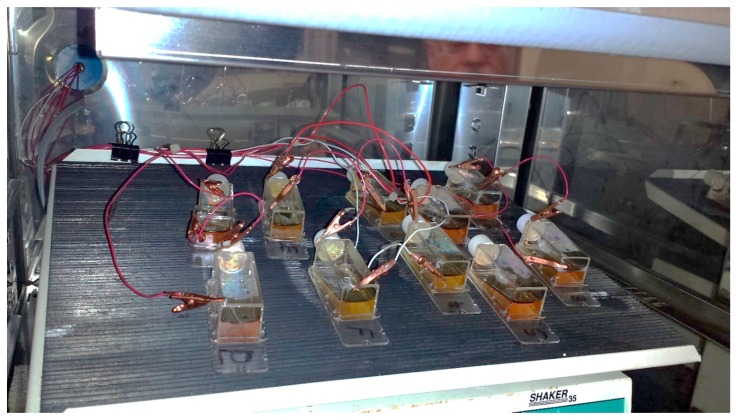
Cell growth flasks during electrochemical oxidative stress treatment. A total of 8 flasks are treated with direct current and 2 control flasks without current are gently rocking inside the cell growth incubator.

**Figure 3:**
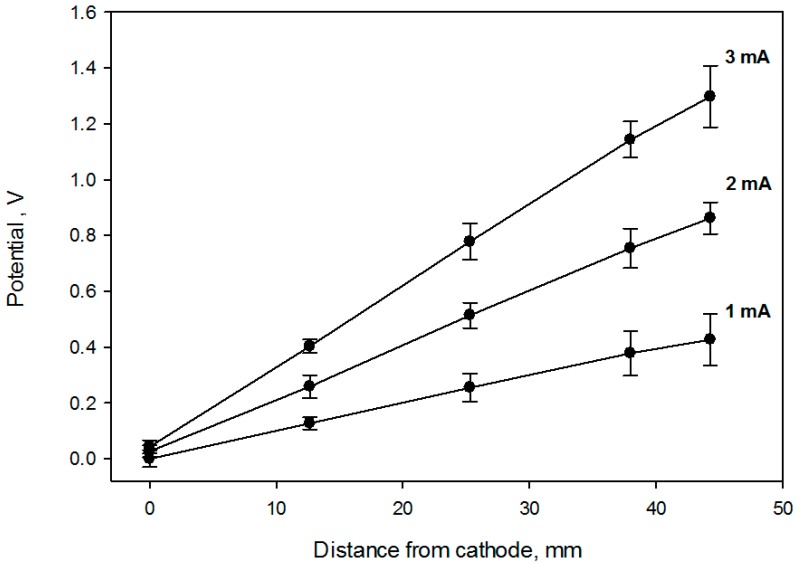
Potential distribution calibration for three flask current values. The solution potential was measured with a reference electrode mounted on a XYZ translator. Error bars represent one standard deviation from 3 independent measurements.

Cells were grown for 72 h (37 °C, 5% CO_2_ and 90% relative humidity) before the application of the potential gradient using a bipolar electrode scheme. At least three replicates were produced under each treatment. Constant current to cell growth flasks inside the incubator was supplied by the potentiostat/galvanostat (Model 363, PAR, Oak Ridge, TN, USA). Flasks were placed on a rocker during the electrochemical treatment providing constant mixing and eliminating pH and/or chemical gradients due to medium electrolysis close to both electrodes (Figure 2) as observed by the uniform color of the pH indicator dye. Prior to cell exposure, the spatial potential distribution in the cell media was calibrated by placing the Ag/AgCl micro-reference electrode (Microelectrodes Inc., Bedford, NH, USA) at different locations along the flask bottom in a separate experiment [26]. Potential values are provided relative to standard hydrogen electrode (SHE). The flask top was detached during calibration, while the reference electrode was mounted on a XYZ translator stage and kept at 0.5 mm distance from the bottom flask surface (Figure 1). A set of potential calibration curves for 3 flask current values is shown in Figure 3. Cell growth conditions were maintained during the potential calibration (37 °C, 5% CO_2_ and 90% relative humidity).

### 2.3. Live Dead Assay

Immediately after the potential gradient application, the growth medium was removed, flasks rinsed twice with Dulbecco’s Phosphate Buffered Saline (DPBS) and live-dead assays (Live/Dead mammalian cell viability/cytotoxicity kit L-3224, Life Technologies, Grand Island, NY, USA) performed according to the manufacturer protocol. The assay uses calcein AM (emission at 515 nm) for live cell stain and ethidium homodimer-1 (emission at 628 nm) for dead. Following 30-min incubation with fluorescent dyes and rinsing with DPBS, the flasks were refilled with DPBS and placed on the microscope stage for imaging.

### 2.4. ROS Assay

Generation of reactive oxygen species was detected with CellRox green assay kit C10492 from Life Technologies, Grand Island, NY, USA. Following their treatment by the electrochemical potential gradient, surface attached cells were gently rinsed with DPBS that was replaced with Dulbecco’s Modified Eagle’s Medium, containing 2.5 mmol/L CellRox dye in the dark. Flasks were imaged after 40-min incubation with green dye using AlexaFluor filter set.

### 2.5. Imaging

**Figure 4.**
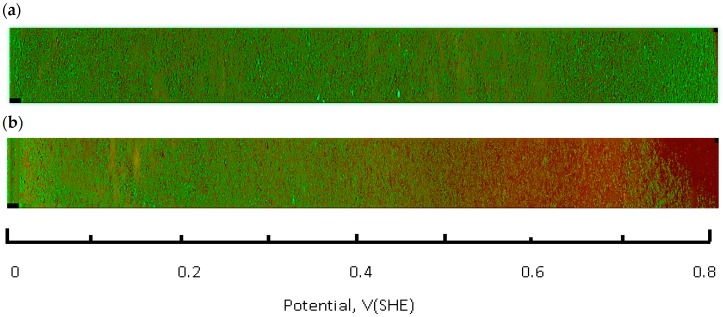

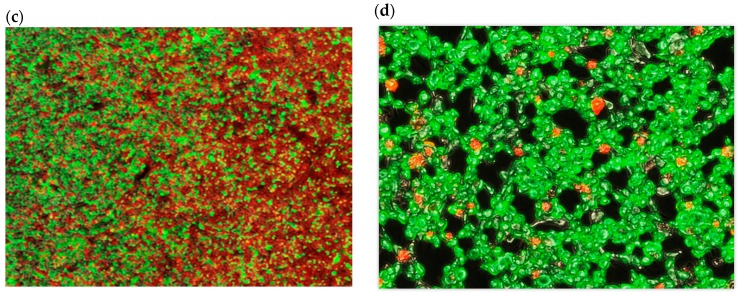
Fluorescence images of the cell growth flask bottom obtained with live/dead stain and plotted alongside the solution redox potential scale (green-live, red-dead). (**a**) control sample, no potential gradient; (**b**) 1 h treatment at 2 mA, both images acquired using 5× objective; (**c**) transition region at 0.42 V, 10× objective; (**d**) 20× objective.

**Figure 5.**
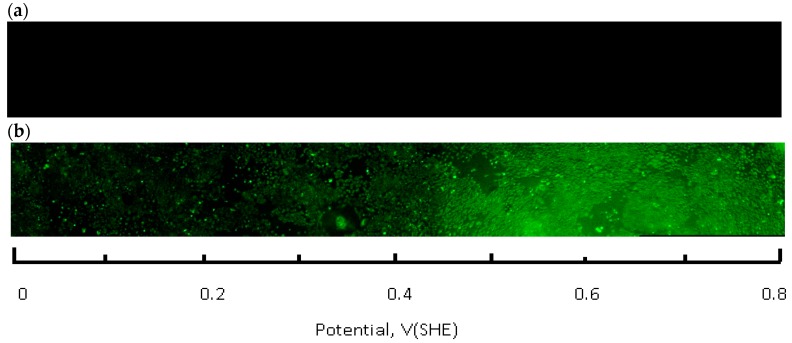
Fluorescence images of the cell growth flask bottom, obtained with a ROS green reagent and plotted alongside the solution redox potential scale. (**a**) control sample, no potential gradient; (**b**) 1 h treatment at 2 mA, both images acquired using 5× objective.

A Zeiss Axio Observer Z1 microscope (Thornwood, NY, USA), equipped with a CoolSNAP HQ2 CCD camera (Photometrics, Tucson, AZ, USA) and Colibri 2 LED (Zeiss, Thornwood, NY, USA) light source was used for image acquisition. A total of 32 areas (frames) were imaged along the full length of the flask bottom using a 5× lens. Each frame covers several hundred CHO cells. The average of three parallel rows was imaged for each flask spaced 3 mm apart. Images were processed and analyzed with ZEN Pro2 (Zeiss, Thornwood, NY, USA) and Image J 1.48 software packages (National Institutes of Health, Bethesda, MD, USA) experiments were conducted at least in triplicate. The frame signals were spliced along the slide length resulting in a full slide live-dead cell image (Figure 4). The fraction of live cells (i.e., cell viability) was calculated by integrating the green (calcein AM) fluorescence channel, followed by normalization against the control flask, which was not connected to the current source. Next, this number (representative of several hundred cells in an integration area) was plotted as a function of the solution redox potential, using location—specific values obtained from the calibration curve, shown in Figure 5. Basic statistical values—means and standard deviations were calculated using the Sigma Plot 12.5 software package (Systat Software, Inc., San Jose, CA, USA). Fractions of live cells, subjected to the electrochemical potential gradient treatment were compared to control sets, obtained from imaging the untreated flask. A Mann-Whitney rank sum test (Sigma Plot 12.5) between the two data groups returned statistical differences with probability values as indicated in figure captions.

## 3. Results

The live-dead fluorescence image of the flask bottom slide shows the CHO cell layer condition following incubation under the redox potential gradient or a fixed time interval (Figure 4). Compared to the control sample that was not connected to the current source (upper panel in Figure 4), the treated sample image develops a gradually changing fluorescence intensity profile (lower panel in Figure 4). The decreasing intensity of the calcein fluorescence, and simultaneously intensifying ethidium homodimer emission from left to right is consistent with an accumulative cell death when exposed to progressively more positive solution redox potential. The cell viability ratio was calculated relative to untreated controls from the integrated pixel density at green and red fluorescence channels with a custom Image J software macro. Increase in ROS concentration, measured in a separate experiment, occurred in the same redox potential range as the decrease in cell viability (Figure 5).

**Figure 6:**
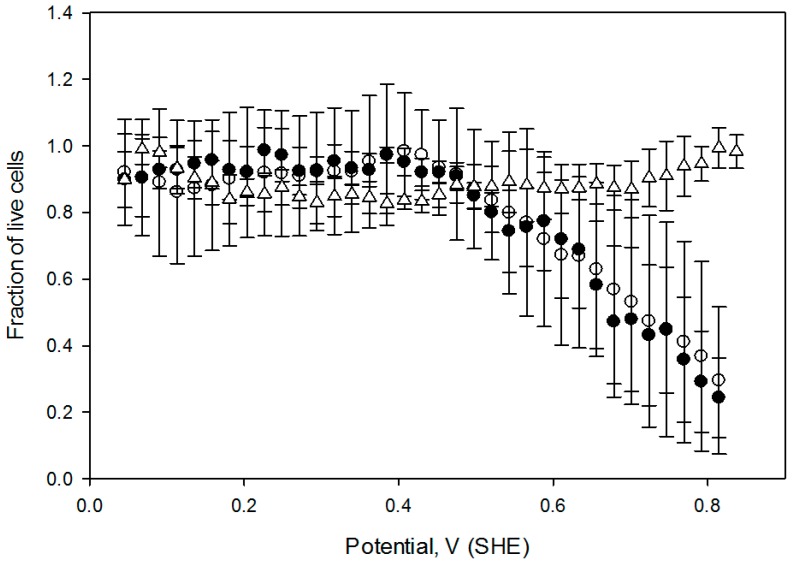
Fraction of live cells as a function of media solution potential. A 2 mA DC current was passed through the growth flask for 1 h (filled circles) and 2 h (open circles), control flask with no current (triangles). Error bars correspond to one standard deviation at *n* = 4. Live cell fractions for 1 h and 2 h treated cells were statistically different from the control at *p* < 0.001 and *p* < 0.002 respectively (Mann-Whitney rank sum test).

The effect of the oxidative treatment on the calculated live cell fraction is shown Figure 6, which contains the cell viability ratio plotted vs. the solution redox potential for 1 h and 2 h treatments at 2 mA DC current. Application of the constant 2.0 mA current imposes a linear redox potential gradient, ranging from 0 V to about 820 mV at the surface attached cell layer (Figure 3). Cell viability is similar to the control for media redox potentials lower than 0.4 V and starts decreasing at higher values. Overlapping live cell fraction profiles at 1 h and 2 h electric treatment duration imply that cell survival under exogeneous oxidative stress is comparable, indicating that redox equilibrium between a cell and media is already established within the first hour. Cell viability vs. solution redox potential dependence, produced when cells are exposed to the electrochemical potential gradient suggests that a two—state transition could be a viable model, with a half maximum effective potential as a numerical characteristic, as shown in Table 1. Such notions are widely used to describe dose-response curves in toxicology [8], and midpoint potential numerical values (Table 1) would be useful to compare cell viability when subjected to oxidative stress in diverse experimental circumstances [27].

**Figure 7:**
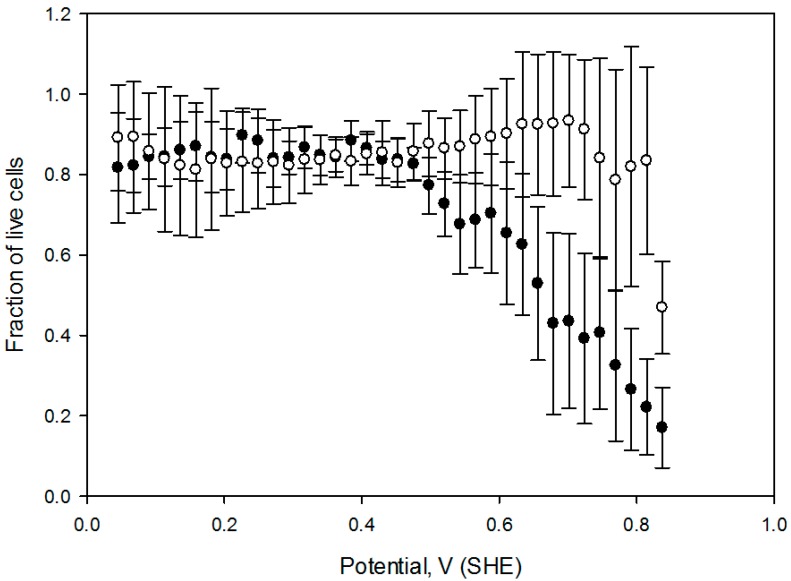
Fraction of live cells as a function of solution potential in media without (filled circles), and with 10 mmol/L GSH (open circles). A 2 mA DC current was passed through the growth flask for 1 h. Error bars correspond to one standard deviation calculated from *n* = 7. Live cell fractions with and without GSH were statistically different from control at *p* < 0.001 (Mann-Whitney rank sum test).

We investigated the effect of the extracellular reduced glutathione on CHO cell viability when subjected to an electrochemical potential gradient. Extracellular GSH is known to increase intracellular GSH concentrations by releasing bound glutathione from mixed disulfides with membrane proteins in some mammalian cells, as a result boosting cellular resistance against oxidative damage [28]. Our data shows that the cell death onset potential increases approximately 200 mV when the cell media is supplemented with 10 mmol/L reduced glutathione GSH during the application of electrochemical treatment (Figure 7). In both experiments, CHO cells were exposed to a similar potential gradient for 1 h. A shift in cell death onset potential was also confirmed when cells were pretreated with 10 mmol/L GSH for 1 h preceding the application of the electric field.

Exogenous OS inducers vary widely in their interaction mode with the live cell. In addition to perturbing the extracellular redox environment, some agents infiltrate cellular membranes and can chemically react with cytosolic or even nuclear constituents. Moreover, they can also affect membrane receptors and disturb cell-signaling pathways [2,11]. Such multimodal action often hampers the mechanistic OS analysis even when the overall reducing or oxidizing power of the compound can be measured using voltammetry [5,8,29]. Definition of the OS strength, however, is not straightforward and cannot be substituted by a simple chemical activity for these reasons.

**Figure 8:**
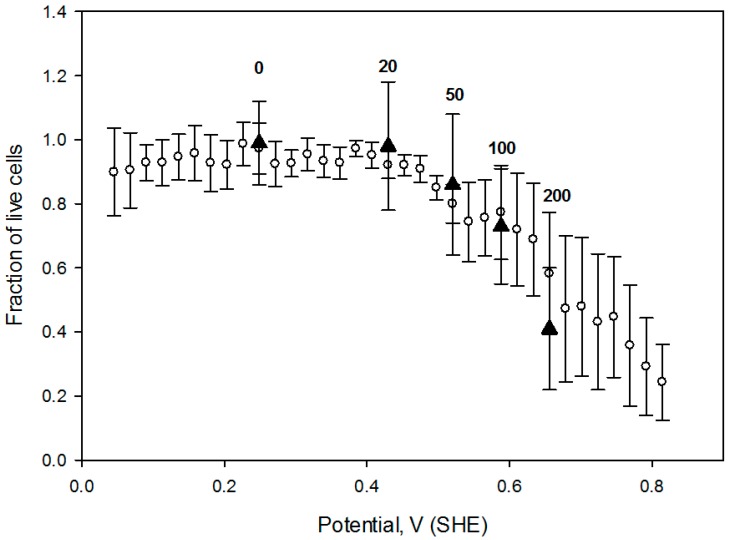
CHO cell viability ratio variation with the solution electrochemical potential in the hydrogen peroxide solutions following 1 h incubation (filled triangles). Numbers on the graph indicate the calculated hydrogen peroxide concentrations, based on dilution in media, in mmol/L. Error bars correspond to one standard deviation, calculated from *n* = 5. Solution potential in peroxide—containing media was assessed using Ag/AgCl reference electrode immediately following peroxide introduction. Open circles represent the live cell fraction, measured under electrochemical potential gradient (data from Figure 6). Live cell fractions for 1 h electrochemically treated cells and incubated with hydrogen peroxide were not statistically different at *p* = 0.713 (Mann-Whitney Rank Sum test).

Therefore, it is worthwhile to compare cell responses to an OS dose (defined as OS strength and the exposure duration), induced by several agents. In this regard, we measured CHO cell survival when exposed for 1h to four hydrogen peroxide concentrations (20 mmol/L, 50 mmol/L, 100 mmol/L and 200 mmol/L) and compared the live cell fraction vs. media redox potential (Figure 8). Redox potential values of these H_2_O_2_ preparations were measured in a separate experiment after hydrogen peroxide was diluted into the cell growth media. Notably, cell viability vs. media redox potential dependence is comparable to the one obtained under potential gradient (open circles in the Figure 8) suggesting that acute exogenous hydrogen peroxide induced CHO cell OS is related to a positive shift in the solution redox potential, at least in this limited concentration range.

## 4. Discussion

In this study we show that an electrochemical potential gradient can serve as a tool to simulate exogenous oxidative stress on surface attached mammalian cells. A simple arrangement permits direct imaging and analysis of cell viability, following exposure to a range of solution redox potentials. The in vitro OS test vessel consists of a cell growth flask, fitted with two Pt electrodes that support a direct current along the flask bottom. The applied potential span and gradient slope can be controlled by adjusting the constant current magnitude across the cell with media potential calibration achieved with a sliding reference electrode. The slide vessel system can be readily adapted for a variety of cellular assays such as ROS detection (Figure 5), apoptosis, etc. Moreover, the cells could be analyzed for oxidative DNA, lipid or protein damage biomarkers using single-cell gel electrophoresis [30] or immuno-histochemical methods [31]. The system is particularly advantageous for providing a stable and linear interval of oxidative and reductive solution potentials in a single experiment. Continuous medium mixing during the electrochemical treatment by placing cell growth flasks on a rocker eliminates media electrochemical byproduct gradients without the use of salt bridges [32], thus avoiding medium and salt interdiffusion. With a multi-channel current source, several cell growth vessels can be used in parallel for replicate testing. The galvanostatic methodology permits the connection of several flasks in series and evens out the potential gradient profile, notwithstanding potential drop variation in the double layers surrounding the electrodes. The vessel contents can be replaced before, during and after treatment in order to test a wide range of solution conditions and cell images that can be acquired during the oxidative treatment. This is particularly beneficial to in vitro testing the effectiveness of additives such as anti-oxidants, as it enables monitoring a real time cellular response. This was demonstrated by a shift in cell viability profile to higher media redox potential values when treated with reduced glutathione (Figure 7). In addition, various cell types including cancer cell lines can be compared in their resistance to oxidative potentials and effectiveness of various drugs [33]. The slide vessel system is easy to sterilize, which is an extra advantage in efficient testing of infectious cells or toxic reagents.

## 5. Conclusions

In conclusion, we believe that this experimental platform will facilitate studies of the oxidative stress characteristics on different types of cells by enabling the generation of reproducible electrochemically controlled redox environments for cultured cells.

## Figures and Tables

**Table 1:** Effective midpoint potential values for live to dead transitions.

Treatment Conditions	Effective Midpoint Potential (mV)
2.0 mA, 1 h	590 ± 20 (*n* = 4)
2.0 mA, 2 h	580 ± 40 (*n* = 4)
2.0 mA, 1 h + 10 mM GSH	790 ± 60 (*n* = 7)

Uncertainties indicate one standard deviation.

## NIST Disclaimer

Certain commercial equipment, instruments and materials are identified in this chapter to specify an experimental procedure as completely as possible. In no case does the identification of particular equipment or materials imply a recommendation or endorsement by the National Institute of Standards and Technology nor does it imply that the materials, instruments, or equipment are necessarily the best available for the purpose. Official contribution of the National Institute of Standards and Technology; not subject to copyright in the United States.
